# Predictors of COVID-19 Vaccine Confidence: Findings from Slums of Four Major Metro Cities of India

**DOI:** 10.3390/vaccines10010060

**Published:** 2021-12-31

**Authors:** Sathyanarayana Tamysetty, Giridhara R. Babu, Biswamitra Sahu, Suresh Shapeti, Deepa Ravi, Eunice Lobo, Chinnu Sara Varughese, Amita Bhide, Avinash Madhale, Mukta Manyal, Mahesh Kamble, Asokananda Konar, Pabak Sarkar, Dipesh Kumar Das, Partha Sarathi Mukherjee, Kultar Singh, Ankur Singh Chauhan, Aditya Naskar, Rajesh Bhatia, Sonalini Khetrapal

**Affiliations:** 1 Indian Institute of Public Health, Bangalore 560023, India; giridhar@iiphh.org (G.R.B.); Biswamitra.sahu@gmail.com (B.S.); SURESHSHAPETI@iiphh.org (S.S.); deepa@iiphh.org (D.R.); eunice.lobo@iiphh.org (E.L.); 2 Seth G.S. Medical College and KEM Hospital, Parel, Mumbai 400012, India; chinnusara@hotmail.com; 3 Tata Institute of Social Science, Mumbai 400088, India; amita@tiss.edu (A.B.); avinash.madhale@tiss.edu (A.M.); muktamanyal@gmail.com (M.M.); kamblemahesh@tiss.edu (M.K.); 4 Liver Foundation, Kolkata 700150, India; asoke.konar@gmail.com (A.K.); pabak.sarkar@gmail.com (P.S.); dkdas1983@gmail.com (D.K.D.); spartham@gmail.com (P.S.M.); 5 Sambodhi Research and Communication, Pvt Ltd., Noida 201301, India; kultar@sambodhi.co.in (K.S.); ankurheathwalk@gmail.com (A.S.C.); aditya.naskar@sambodhi.co.in (A.N.); 6 Asian Development Bank, New Delhi 110021, India; Drrajesh.bhatia1953@gmail.com (R.B.); skhetrapal@adb.org (S.K.)

**Keywords:** vaccine confidence, COVID-19, urban slum

## Abstract

There are limited studies on COVID vaccine confidence at the household level in urban slums, which are at high risk of COVID-19 transmission due to overcrowding and poor living conditions. The objective was to understand the reasons influencing COVID-19 vaccine confidence, in terms of barriers and enablers faced by communities in urban slums and informal settlements in four major metro cities in India. A mixed method approach was adopted, where in field studies were conducted during April–May 2021. First, a survey of at least 50 subjects was conducted among residents of informal urban settlements who had not taken any dose of the COVID-19 vaccine in Mumbai, Bengaluru, Kolkata and Delhi; second, a short interview with five subjects who had taken at least one dose of the vaccine in each of the four cities to understand the factors that contributed to positive behaviour and, finally, an in-depth interview of at least 3 key informants in each city to ascertain the vaccination pattern in the communities. The reasons were grouped under contextual, individual/group and vaccine/vaccination specific issues. The most frequent reason (27.7%) was the uncertainty of getting the vaccine. The findings show the need for increasing effectiveness of awareness campaigns, accessibility and the convenience of vaccination, especially among vulnerable groups, to increase the uptake.

## 1. Introduction

Vaccines have achieved considerable success in maintaining the health of the population across cultures and nations. From time to time with the effective use of vaccines, large numbers of people have benefited in being free from vaccine-preventable diseases. Nearly two to three million deaths are prevented each year since the advent of immunization programmes, and vaccines are cost-effective preventive care [1]. As per the Centre for Disease Control (CDC), vaccines are among the top ten effective public health interventions of the twentieth century [2]. Vaccines have a significant benefit in terms of reduction of treatment cost, and enhance the productivity of the workforce in the long term [3]. A crucial point is with regard to perspectives that may hinder the decision at the individual or family level related to the COVID vaccines. However, we cannot reject the benefit of vaccines in protecting us from infectious diseases. Nevertheless, we also cannot assume that the availability of the COVID-19 vaccine is sufficient to vaccinate all of the target population. Vaccination has to be free from fears and negative perspectives, which may halt the speed and size of vaccination coverage.

Hence, COVID vaccination hesitancy needs to be understood in a particular socio-cultural context while examining differing perspectives (experts, civil society members, community groups) at different levels and their use of different perspectives (multiple realities) in their belief to decide to either get for COVID-19 vaccination or not.

### 1.1. Vaccine Confidence Measurement Models

The SAGE working group on vaccine hesitancy (Hesitancy word appears to send negative image, therefore, hereafter this paper uses vaccine confidence instead of vaccine hesitancy) has defined the term ‘vaccine hesitancy’ as a delay in acceptance or refusal of vaccination despite the availability of vaccination services. Vaccine hesitancy is a complex behaviour and context-specific. It differs across time, place, person and vaccines [4]. It is a spectrum where an individual can express acceptance of vaccine with certainty, have vaccine confidence, delay vaccination, be unsure of vaccination, refuse vaccines or fall anywhere in between. The protection motivational theory believes in threat appraisal (perceived severity of health threat/individual vulnerability) and coping appraisal (perceived self-efficacy) [5]. Recognizing both threat appraisal and coping appraisal and defining vaccine confidence is important to improve good vaccine coverage. Furthermore, the factors that influence vaccine confidence have been clearly expressed through the ‘3 Cs’ model of determinants—confidence, complacency and convenience, and have been included in the definition [6]. The recommendation by the SAGE committee has important take-aways for increasing the understanding of vaccine hesitancy, determinants and challenges, for improving acceptance and sharing lessons from multiple country contexts which are critical for the development of new tools for addressing factors that influence vaccine confidence [7].

Furthermore, the Working Group has developed a Vaccine Hesitancy Determinants Matrix on influencers of vaccine confidence, as it was realized that defining alone was not enough, but an understanding of the risk factors was crucial in implementing appropriate interventions to increase vaccine uptake. Thus, the influencing factors have been grouped under contextual, individual/group specific and vaccine/vaccination specific factors as shown in Table 1.

*Contextual influences* deal with socioeconomic, environmental, political, cultural, economic and institutional factors. *Individual/group-specific factors* influences arise from personal beliefs about the vaccine and peer environment. *Vaccine/vaccination-specific influences* are directly related to vaccines or vaccination [6]. We applied the SAGE model of the vaccine hesitancy determinants matrix to examine the vaccine confidence issues in our study.

### 1.2. Context of COVID-19 and Urban Slums/Poor

In India, more than 100 million residents reside in urban slums [8]. The official estimate shows the number of slum dwellers increased in India. The unprecedented rate of urbanization led to the rise of informal settlements or slums. Slums are characterized by the lack or limited health, education services, limited living spaces, insecurity and informal jobs or no jobs [9]. Compared to the rest of the population, slum dwellers have a higher proportion of COVID-19 related morbidity and mortality [10], and their vulnerability increases due to economic shocks and several depend on informal income.

Furthermore, the availability of a safe and effective vaccine is essential to control COVID-19. Currently approved vaccines against COVID-19 have shown promising results. However, adequate coverage of eligible population depends on the uptake of the vaccine, which may vary across sub-populations such as the slum population [11], ethnic minority population [12], and healthcare workers [13]. The challenge of improving vaccine confidence among the general public has become a hindrance for immunization programmes in achieving good vaccine coverage [14]. This eventually leads to an increased risk of failure to prevent disease transmission and a surge in vaccine-preventable diseases. In this context, the World Health Organization (WHO) has highlighted the lack of vaccine confidence as one of the top 10 threats to global public health in 2019 [14]. Therefore, an adequate understanding of differing perspectives, barriers and enablers to COVID vaccine uptake could potentially give insights for developing interventions focused on improving vaccine uptake. This is specifically important considering evidence that the urban poor or slum populations are more likely to be affected by COVID-19 and experience higher morbidity and mortality [10]. Additionally, previous study reviews recognise the growing trend of varying concerns against COVID-19 [11] signifies the necessity of monitoring of vaccine confidence [15]. As a result, the focus of this study was to examine the factors that influence to receiving of the COVID-19 vaccine in urban slums in India.

This paper aims to explore the enablers and the barriers of vaccine acceptance among the urban poor population in four major metro cities of India. As the health care system geared up to undertake the COVID-19 vaccine delivery, the results of this study are expected to provide the necessary information to modify or re-adjust the existing strategies. Furthermore, there is an urgent need to overcome the key barriers experienced by the urban poor population using evidence-based intervention to ensure equitable COVID vaccine coverage [16,17].

## 2. Materials and Methods

This study adopted a mixed-method approach to examine the factors that influence vaccine confidence in urban India. The objective is to understand the reasons for lack of vaccine confidence, barriers, and other challenges to COVID-19 vaccination faced by communities in urban slums and informal settlements in Bengaluru, Delhi, Kolkata and Mumbai. The field studies were conducted using a pre-decided and validated design. Four city-specific partners simultaneously undertook the study during April–May 2021. The study employed both quantitative and qualitative methods to examine barriers and facilitators for vaccine acceptability and factors contributing to vaccine confidence in vulnerable urban poor slum populations who are at high risk for COVID-19 infection through the following approaches.

First, a quantitative survey of convenience was undertaken from a sample of 50 subjects from informal urban settlements who had not taken any dose of the COVID-19 vaccine in Mumbai, Bengaluru, Kolkata and Delhi. Second, we interviewed five subjects who have taken at least one dose of the vaccine in each of the four cities to understand the factors that contributed to the positive behaviour. Finally, an in-depth interview of three key informants in each city on vaccination patterns in the communities was conducted.

### 2.1. Locations

The study sites were the following urban slums in four major metro cities of India, as shown in Table 2.

### 2.2. Process of Data Collection

#### 2.2.1. Data Collection

With the help of community leaders working in the area, we selected 50 individuals by convenient/purposive sampling. The data was collected from the selected individuals while visiting house to house in Bangalore, Mumbai and Kolkata. In Delhi, the data was collected telephonically due to the lockdown. A total of 296 samples were collected from all four locations using the survey method.

##### Context of Sampling

The background regarding the decisions that were taken in the beginning of data collection to suit the appropriateness of the survey sample size and method of data collection into perspective. The task that we had set out was a rapid understanding of vaccine hesitancy across informal settlements in four major urban cities of India. The data was collected during the peak of the second wave of COVID infection. The spikes in the COVID situation were dynamic and varying across all four sites. However, the evidence was to be collected within a quick turnaround of one week of data collection because time was required for data processing and analysis for generating speedy policy recommendation, albeit without missing out on contextual reality. It was mutually decided that a sample of a minimum 50 respondents was feasible. Hence, we used a convenience sampling procedure for conducting the survey considering what was feasible under these exceptional circumstances. The Bangalore team was able to recruit a bigger sample due to relatively lesser operational challenges. However, data collection in Delhi was conducted telephonically because there was high-risk environment of COVID-19 and the inability of the team to be present in the field at the time of data collection.

#### 2.2.2. Ethical Approval

The ethical approval for the parent study was taken from respective institutes in all four cities.

## 3. Results

The results section is structured as follows. First, we describe the profile of study respondents (interviews and survey). Second, we describe relevant quantitative survey data along with qualitative explanations. We applied the SAGE model of the determinant of vaccine confidence in our data analysis and presentation such as (a) contextual influences; (b) individual/group influences and; (c) vaccine factors influence.

The background of in-depth interview participants, key informant interview participants and survey participants of Mumbai, Bengaluru, Kolkata and Delhi is detailed in the following tables.

### 3.1. Socio-Demographic Profile of In-Depth Interview and Key Informant Interview 

The socio-demographic and vaccination profile of in-depth interview participants from Mumbai, Bengaluru, Kolkata and Delhi is detailed in Table 3.

The socio-demographic, designation and work experience profile of key informant interview participants from Mumbai, Bengaluru, Kolkata and Delhi are detailed in Table 4.

### 3.2. Socio-Demographic Profile of Survey Participants

The socio-demographic, employment and household income profile of survey participants is detailed in Table 5.

Of all the respondents in this study, 42% belonged to the 45–50 years age group, followed by 17% from 51–55 years and 14% from 56–60 years. Fifty-seven percent were women.

Fifty-three per cent were unemployed at the time of the field survey and the remaining who worked were employed in the informal sector. Yet, most of them had meagre incomes, with 73 per cent reporting a monthly household income of less than Rs 20,000. 

Additionally, basic demographic characteristics such as age and gender were cross-tabulated with trust over the vaccine (see Table 6). Also, a chi-squared test was conducted in order to assess where these background characteristics have any bearing over trust in the vaccine.

The results indicate that trust in the vaccine is slightly higher among populations of 60–80 years of age. However, there is no statistical association between people’s trust in the vaccine and their gender or age.

### 3.3. Factors Influencing COVID-19 Vaccination

The reasons for vaccine confidence have been presented below, after survey of 296 study participants. WHO’s ‘3C model’ has been used to express the various factors. The majority of the reasons are related to individual and group influences, followed by contextual influences. Vaccine/vaccination specific issues were comparatively reported less frequently, except for the uncertainty of getting the vaccine. The field surveys generated data on responses of various people in the urban slums on factors that could influence the vaccination behaviour of these communities. The factors are shown in Table 7.

The prime reasons for not getting vaccinated, as reported by the respondents are complications and/or futility of getting vaccinated, uncertainty of getting vaccine, fear of getting vaccinated, and lack of understanding of safety of vaccine and AEFI. The Chi-square test reveals that 10 of the 16 reported reasons for non-vaccination is statistically associated with respondent’s place of residence (city), indicating the heterogenity in the responses.

### 3.4. Contextual Influences

Around one-fifth of respondents from Delhi and Mumbai were dependent on others/families in deciding to vaccinate, whereas it was less than 10% each in Bengaluru and Kolkata. Similar proportions reported dependence on family/others to register in the four cities. The inability to spare a day from work was the most frequent reason in Bengaluru (13%), while in Delhi it was 13%. Vaccination centres being stated as too far was reported more in Delhi (9.3%). Physical disability was an issue in Bengaluru (10.2%), Mumbai (<10%) and Kolkata (8%). Lack of supporting documents was most frequently reported in Bengaluru (14%), while in the other three cities, it was less than 10%.

### 3.5. Qualitative Findings

Qualitative data provide additional insights into the relation between reasons for vaccine confidence patterns and likely low/high coverage. The findings are arranged under sub themes, namely, gender, geographical, technological and socio-economic barriers (see Table 8, Table 9, Table 10 and Table 11).

Gender: Family dynamics and gender played a role in vaccine confidence. As a key informant put it, the need for women to depend on their husbands and family to decide to get vaccinated was a potential barrier. Family and neighbours were influential in terms of sharing of information, decision making, support and accompaniment to the vaccination centre.

Geographical barriers: Distance of the vaccination centre and accessibility was a factor in deciding to get vaccinated. Around 9.5% of respondents from the survey stated that the vaccination centre was far. Some people were not aware of the hospitals where the vaccination drive was taking place.

Technological barriers: 21.4% of respondents from the survey said they were unable to register. Multiple factors like good network connectivity, digital literacy, possession of a good smartphone were lacking in the urban slums, as mentioned by the key informants. Hence, most of the registrations were done on-site.

Socioeconomic reasons: Most people in the slums were daily wage earners and were hesitant to take off and accompany the dependents in their families to the vaccination centre. This would mean losing their daily income. Different strategies are required to be vigorously implemented by the government to tackle this problem.

### 3.6. Individual/Group Influences

Mumbai had the greatest number of respondents stating lack of faith in immunization (20%), followed by Delhi (7.4%) and Kolkata (6%). Again, maximum study participants in Mumbai (44%) felt complications and/or futility of getting vaccinated as the reason for vaccine hesitancy, followed by Delhi (37%). Lack of understanding of the safety of vaccines and side effects were mostly reported in Delhi (35.2%) and Mumbai (22%). Thirty-eight percent of respondents in Mumbai and 14.8% in Delhi had fear of getting vaccinated. Twenty-two percent each in Mumbai were doubtful about the utility of the vaccine in containing the pandemic, and did not believe in the government system.

Group influences: Peer influences had a major role in shaping the opinions of the people. Some community members were hesitant to get vaccinated due to cautioning by a family member, death of a popular media personality due to AEFI from vaccination, while others were motivated by their friends, neighbors, community volunteers, employer, colleagues and the media. The theme of group influence is illustrated in Table 12.

Individual influences-misinformation/rumours: Certain people have misconceptions regarding COVID-19, vaccination and the impact of comorbidities. For example (see Table 13), a community member stated that he does not feel the need to vaccinate since he does not have COVID-19. Another member was not convinced about the efficacy of the vaccine and did not want to take the risk, since he came to know that he could contract COVID-19 even post-vaccination. One community member was motivated by his manager to get vaccinated despite conflicting advice from other people. 

Knowledge and awareness about AEFI at individual/family level and trust: In-depth interviews with the key informants have also revealed that some people were very concerned about the safety of the vaccine and AEFI. They were not only concerned about the immediate side effects but rumors of longer-term health complications were also rife. Also, refer to the verbatims of this theme below in Table 14 below.

Experience with past immunization: Successful immunization with no AEFI by peers and healthcare workers was seen as an enabler for people to have vaccine confidence. Key informants expressed the need for repeated communication through various channels to address the fears of the public (refer to Table 15 for details).

### 3.7. Vaccine/Vaccination Specific Influences

Uncertainty around getting the vaccine was seen most frequently among respondents in Kolkata (48%) and Delhi (24.1%). Ten percent or fewer respondents cited inappropriate timing as a factor in all four cities. The inability to register was an issue in Bengaluru (26.3%) and Mumbai (20%). Other reasons, like fear of overcrowding at the site, fear of symptoms, and poor knowledge of the process, were reported in Mumbai (24%) and Delhi (18.5%). When observing the most common reasons for poor vaccine acceptance in the individual cities, Bengaluru reported the highest percentage that indicated an inability to spare a day from work (13%), while Delhi (13%) and Mumbai (2%) reported complications and/or the futility of getting vaccinated and Kolkata reported uncertainty in getting the vaccine (48%). 27.7% from the field survey reported uncertainty in getting the vaccine. Two reasons have been found to be responsible for the uncertainty—vaccine supply and access.

Vaccine supply issues: A community member has mentioned that people were not sure if the vaccine would be available at the centres. Some were sent back by the hospital due to a lack of stock. This has resulted in the delay in vaccination. However, the key informants are of the view that people are still willing to take the vaccine despite these issues. Table 16 delineates the vaccine supply issues as expressed by the key informants of this study.

Access issues: Some barriers to getting vaccination were the lack of knowledge on the process, where to go and how long to wait, hesitancy to wait in long queues and overcrowding. Enablers were the presence of transport, the absence of a waiting period, the lack of rush and the overall smooth conduct at the centre. Table 17 illustrates access barriers as elaborated by the study participants.

#### Key Barriers and Enablers

The above interview and survey findings have been summarized in the form of barriers and enablers. The vaccination enablers/facilitators included availability of the proper information, improved access to facilities, external support from neighbors, family, and employers and inherent motivation of people that drive them to get vaccinated. Finally, the composite barriers and enablers to vaccine uptake are illustrated in Table 18.

## 4. Discussion

This study, through a mixed-method approach, explored the factors behind the level of vaccine confidence in urban slums in four major cities in India.

### 4.1. Contextual Influences

Within contextual influence, the gender-culture aspect seemingly played a crucial role in the decision to go for vaccination. The study found that the main reasons were dependence on others/family to make the decision to get vaccinated (17.6%) and being unable to register for vaccination (21.4%). Here, gender disparity is being implicated. Mostly, women are known to be dependent on family for decision making. Similarly, a nationwide study from Qatar observed relatively higher hesitancy and resistance among the female gender [18] [, whereas a in a study from New York city, females with chronic diseases have a lower likelihood of getting the vaccine [12]. In a study done by Echoru et al., men were found to have more acceptance than women, albeit for a different reason, but that women had more fear of vaccination [19]. The dependency on others to go for vaccination also deters people from vaccination. Often women in the slums and informal settlements need to get permission from their husbands or other family members and are dependent on their decisions and/opinions to get vaccinated. Even for routine child immunization, women had to depend on husbands or elders for taking the decision, as is found in research work done in Nigeria [20]. Dependence on others to register for vaccination in urban slums may be linked to the general lack of digital literacy in the population. Other factors found in our study were the inability to spare a day from work (6.4%), the distance of the vaccination centre (5.7%), physical disability or health problems (2.7%), and lack of supporting documents (0.7%). Geographic barriers prevented proper utilization of immunization services [20].

Besides these issues, domestic chores also make it difficult for them to take the time and go out for vaccination. Many people were also found to be dependent on daily wage labour and cannot afford to miss a day at work, especially during these times when financial security for most households is uncertain. Due to the nature of their work, and the lack of time away from it, they find it difficult to go and get vaccinated. Furthermore, they are also unable to accompany aged members in their family for vaccination. The urban population, especially the slum population, have a unique set of factors that expose them to increased risk for COVID-19 infection [11]. They also consist of a migrant population whose health parameters are poorer than the rest, due to low socioeconomic background, limited access to social welfare and vulnerability to marginalization. Socio-economic vulnerability has been determined to be a factor of low vaccine intention [21].

### 4.2. Individual and Group Influences

The most frequent reason overall for not getting vaccines was lack of confidence in whether the vaccine will protect against COVID-19 (11.3%) Next to this, 28.6% of respondents were concerned about the complications and/or futility of getting vaccinated. One-fourth of the participants felt a lack of understanding of the safety of the vaccine and its adverse effects, while 24% were fearful of getting vaccinated. 11.8% were doubtful about the utility of the vaccine in containing the pandemic. These findings are in agreement with those of previous works on migrants and in low-income countries [22,23], which reported a lack of confidence in vaccines and fear of side effects as the major reasons. A study from Italy found that lack of vaccine safety and concerns about adverse effects are key reasons for vaccine hesitation [24] These reasons may be more relevant in this context as COVID vaccines are relatively new and are produced within a short period of time. Our qualitative data reveals that several individuals in slums do not understand the effectiveness and the need to get vaccinated. Many do not trust the vaccines yet and the effectiveness of these in curbing the pandemic. Some consider that the vaccination might be futile.

Peer influence also had a major role as seen in the results of the qualitative component of our study. Family members, community health volunteers, NGOs, employers, and healthcare workers all contribute to the spreading of correct information. The role of healthcare workers is a recurring subtheme in the interviews. In a study in Nigeria, healthcare workers were reported to be the commonest and most important source of information [19].

Another belief that prevents people from getting vaccinated is their fear of developing health complications and life-long side effects. As per the interviews, it has become apparent that many individuals are swayed by the misinformation being spread on social media about the harmful effects of the vaccine. A study from Poland observed that fear of complications was a reason to avoid vaccination [25]. Experiences with past immunization could produce either positive or negative outcomes, depending on the type of experience [19]. In our study, interviewees have reported a positive experience being the reason for others to get the vaccine. Similarly, In Australia, either the presence of the COVID-19 virus or lockdown had a positive influence to go for vaccination [26].

However, rumours from different sources (social media) seem to have major implications in their perceptions. Our study findings show that the community strongly believe in those misconceptions about the adverse effects of the vaccination that they get from social media groups. The word-of-mouth campaigns of the vaccines in slum areas also hinder people from getting vaccinated. Most of them have been fearful of getting fever, body aches, losing consciousness and feeling nauseated, among others. Other discrete misnomers prevalent among them included risk of death, the adverse effect of the vaccine on the ability to conceive children, and COVID-19 like complications on taking the vaccine. Key informants strongly feel that such false information and paranoia actively dissuades individuals from being vaccinated. Although social media has both a negative (comments) and positive effect (frequently positive) [27], it plays a crucial role in the individual’s perception of the vaccine, and their subsequent action related to uptake of the vaccine. Concerns about the possible complications and safety of the vaccine and a general lack of understanding are limitations to vaccine confidence. Interventions are required to monitor social media [27] targeting of a particular factor; for instance, more effective communication, involving all stakeholders, to convey the right information about vaccines to the urban slum population and increase their faith in immunization. Also, in order to improve the readiness for vaccine acceptance, the Guide to Tailoring Immunization Programmes (TIP) is likely to provide useful tools for identifying vaccine hesitant population subgroups and recognizing the demand- and supply-side immunization barriers and enablers and, finally, to design evidence-informed responses [28].

### 4.3. Vaccine/Vaccination Specific Influences

One of the key reasons for the lack of vaccine confidence is the lack of information regarding where and when to get vaccinated as the information is not widely available to all. 27.7% of respondents reported the reason as the uncertainty of getting the vaccine. Other reasons cited were inappropriate timing (10%) and the inability to register (21%). The shortage of vaccines and uncertainty is an issue not directly related to vaccine hesitancy, but may still result in a delay in vaccine uptake [19]. 8.7% of respondents had a lack of belief in the government system, stating that they will get the vaccine once it’s available in the free market. Other studies support the similar view that mistrust in the government is linked with vaccine supply and service concerns [20,29,30].

In particular, the confusion and complexities in terms of location, schedule and availability of vaccines dissuades people from planning and going out for vaccination, as people find vaccination centres inaccessible and the process inconvenient. There is a lot of confusion in older age groups and women in terms of location, registration process, and other aspects of information. This uncertainty of not getting vaccinated possibly in the nearest centre and the inconvenience of going to distant places seems to deter vaccine uptake.

Finally, the key barriers from our study findings are the inability to spare time due to their economic vulnerability, low digital literacy, misinformation/rumours, inaccessibility to old age groups, inconvenient timing, and fear of adverse effects. Barriers from other studies are fear of side effects (41.2%) and lack of confidence in vaccine effectiveness (15.1%) [31]. The enabling factors from other studies are knowledge of COVID-19, worry/fear regarding COVID-19, higher income, younger age, and testing negative for COVID-19 [31]. The key enabling factors from our findings are better access to vaccination centres, easy registration process, availability of an adequate vaccine, employers highlighting its importance and a positive experience post-vaccination.

### 4.4. Study Limitations

The results were drawn from a purposefully selected informal settlement in four metros. Hence, the findings may not be generalizable to other populations. The increase in COVID-19 cases and shortage in vaccines in metros may also have influenced the respondents. The findings from this study are largely representative and indicative of issues and concerns raised by individuals residing in urban poor resettlements. Nevertheless, the perception of communities has been dynamic and their vaccine confidence level depends on the progression of the pandemic and the advances in the vaccination processes.

### 4.5. The Following Are Key Recommendations

Improvement in vaccine confidence could be tackled on three fronts—information/awareness, improving accessibility, and facilitation and outreach for special segments. In the context of voluntary vaccination by the urban poor, it is equally important to keep the financial costs of getting vaccinated to a bare minimum.

In order to dispel the misinformation that leads to decline in vaccination intent [32], there is need for the development of an effective communication strategy to create trust in the communities, raise awareness, and reveal myths regarding safety and efficacy of vaccines through consistent and evidence-based messages [33]. There is a need to take up vigorous awareness campaigns, telephonic messages, and pamphlets which describe the safety of the vaccine and the active engagement of NGOs [34] and community leaders/champions/employers and community associations [35]. This social mobilization should be utilized to disseminate correct and timely information over social media [36], newspapers, advertisement campaigns, and billboards to increase awareness. This knowledge, which will invigorate demand for the vaccine, needs to coincide with better access through greater awareness of vaccination centres. For building trust on the vaccine, transparency of Adverse Events Following Immunization (AEFI) is critical [37]. Consequently, there is a need to develop and implement a protocol for AEFI response, with a focus on post-vaccination follow-up and communication to handle any adverse events. Furthermore, involving community mobilizers and frontline workers to build trust and engage communities via community engagement practices should be brought into practice [38]. The identification of traditional vaccine-hesitant and resistant areas/groups from local communities has yielded success in the uptake of polio vaccination drives in the past [39]; hence, similar strategies can be employed to build community trust and acceptance in such pockets. The quality of vaccine dispensing service can greatly improve by improving accessibility and friendly delivery of vaccination [33]. Also, the effective distribution of vaccines has to be ensured in order to avoid overcrowding at the vaccination centers [40], and also the internal layout and organization of movement in the centers to reduce crowds, smooth the flow of people and maintaining their safety. In order to ensure equity there is a need to overcome technological disparity [41] by developing systems that ensure easy access of the urban poor to the electronic registration system. Furthermore, access to vaccines is hampered when there are few vaccination centres [42]; therefore, access can greatly improve when there is greater concentration of vaccination centres in close proximity to settlements. Though there are challenges faced by hospitals in sharing public health informatics [43], the need for increased participation by local health facilities (public and private) to provide information on vaccine availability, registration, and vaccination schedules is urgent. In order to overcome opportunity costs faced by the poor in getting vaccinated during weekdays, mass vaccination drives over the weekend can be organized [44]. As part of an inclusive vaccination programme, the WHO and UNICEF policy brief suggests arranging of transport, especially for the disabled, to the vaccination centers [45], and the same can be arranged for the elderly. Specifically, the elderly are more likely to have co-morbidities, and in assessing the correlation between co-morbidities and vaccination it is essential to conduct a post-vaccination follow-up for the elderly.

## 5. Conclusions

The reasons for the lack of vaccine confidence are complex and shaped by varying situations. Our study findings were found to be useful to consider for proactive measures to alleviate key concerns. Furthermore, it is essential to ensure adequate vaccine supply, quality service delivery, optimal timing and appropriate information. There is a need for concerted national, state and local level efforts to understand, analyse and address vaccine confidence at regular intervals. Enhancing understanding in the urban poor/slum population while engaging community-based organisations, strengthening local capabilities to mobilise diverse communities by addressing the community-specific vaccine concerns/reasons from time to time through effective culturally appropriate, tailored messages in vernacular languages supported by ever-emerging evidence could improve coverage of the COVID vaccine. The findings indicate the need to plan and carry out specific result-oriented interventions using key findings such as effective risk communication, which caters to the community’s apprehensions and building trust; and using existing technologies to reach target audiences.

## Figures and Tables

**Table 1:** Vaccine confidence measurement model.

Contextual Influences	Influences Arising due to Historic, Socio-Cultural, Environmental, Health System/Institutional, Economic or Political Factors
Individual and group influences	Personal perception of the vaccine influences of the social/peer environment Personal, family and/or community members’ experience; Beliefs, attitudes about health and prevention; Knowledge/awareness; Health system and providers-trust and personal experience; Risk/benefit (perceived); Immunisation as a social norm vs. not needed/harmful
Vaccine/vaccination specific issues	Directly related to vaccine or vaccination Risk/Benefit; Introduction of a new vaccine or new formulation or a new; recommendation for an existing vaccine; Mode of administration; Mode of delivery; Reliability and/or source of supply of vaccine; vaccination equipment; Vaccination schedule; Costs

Source: [6].

**Table 2:** Showing study locations.

City	Study Area	Partner
Bengaluru	Vijinapuram Slum	Public Health Foundation of India, Bengaluru, India
Delhi	Madanpur Khadar JJ Colony, Nizamuddin Basti slum area and Pochanpur Village, Dwarka	Sambodhi Research & Communications Pvt Ltd. Noida, India
Kolkatta	Borough 12 of Kolkata Municipal Corporation, (southern part)	Liver Foundation = West Bengal, Kolkata, India
Mumbai	Ward M-East, Mumbai Municipal Corporation	Tata Institute for Social Sciences, Mumbai, India

**Table 3:** Socio demographic profile of in-depth interview of community members from Mumbai, Bengaluru, Kolkata and Delhi.

Location	Mumbai (*n*)	Bengaluru (*n*)	Kolkata (*n*)	Delhi (*n*)
Age in years range	52–80 years (5)	51–68 (5)	51–85 (5)	49–61 (5)
Sex	Male (2)	Male (3)	Male (3)	Male (1)
	Female (3)	Female (2)	Female (2)	Female (4)
Religion	Hindu (5)	Hindu (4)	Hindu (4)	Hindu (5)
	Muslim (0)	Muslim (1)	Muslim (1)	Muslim (0)
Education in years (range)	No formal education (3)	No formal education (0)	No formal education (4)	No formal education (3)
	<9 years (0)	<9 years (4)	<9 years (0)	<9 years (2)
	10–12 (2)	10–12 (1)	10–12 (0)	10–12 (0)
	>12 years of education (0)	>12 years of education (0)	>12 years of education (1)	>12 years of education (0)
Vaccination status	Yes (5)	Yes (5)	Yes (5)	Yes (5)

**Table 4:** Key-informant interview participants profile from Mumbai, Bengaluru, Kolkata and Delhi.

Location	Mumbai (*n*)	Bengaluru (*n*)	Kolkata (*n*)	Delhi (*n*)
Age (range)	29–60 (3)	44–55 (6)	38–47 (3)	23–37 (3)
Sex	Male (3)	Male (4)	Male (1)	Male (1)
	Female (0)	Female (2)	Female (2)	Female (2)
Designation	District level officers (3)	District level officers (5)	District level officer (1)	Health officers (2)
	NGO (2)	Clinicians (1)	NGO (1)	NGO (1)
			Volunteer (1)	
Experience	<5 years (1)	<5 years (0)	<5 years (1)	<5 years (2)
	6–20 (0)	6–20 (6)	6–20 (2)	6–20 (1)
	>21 years (2)	>21 years (0)	>21 years (0)	

**Table 5:** Socio-demographic profile of the survey study participants.

Serial Number	Demographic and Socio-Economic Profile	Percentage
1	Age	
	45–50	42 (125)
	51–55	17 (50)
	56–60	14 (40)
	>60	27 (81)
2	Gender	
	Female	57 (169)
	Male	43 (127)
3	Employment status	
	Unemployed	53.4 (158)
	Employed	48 (138)
4	Monthly family income	
	<20,000	73 (214)
	>20,000	27 (82)

**Table 6:** Percentage distribution of Age and Sex with trust over vaccine.

Respondent’s Profile	Trust on Vaccine (%)		*p*-Value
Sex	Yes	No	
Male	46.2	53.8	0.237
Female	43.3	56.7	
Age (in years)			
45–50	43.2	56.8	0.336
51–55	46.0	54.0	
56–60	42.5	57.5	
61–65	32.4	67.6	
66–70	63.0	37.0	
71–80	55.6	44.4	
80+	50.0	50.0	

**Table 7:** Showing major reasons that influence vaccination acceptance.

S No	Reason	Reasons for Not Getting Vaccinated (%) ^1^					Significance (*p* Value)
		Bengaluru	Delhi	Kolkata	Mumbai	Total	
		N = 142	N = 54	N = 50	N = 50	N = 296	
1	Complications and/or futility of getting vaccinated	1.7	37	2	44	28.6	0.000
2	Lack of understanding of safety of vaccine and AEFI	2.7	35.2	12	22	24.3	0.044
3	Uncertainty of getting vaccine	4	24.1	48	6	27.7	0.000
4	Dependence on others/family to make decision to vaccinate	7.7	20.4	4	20	17.6	0.054
5	Dependence on others/family to register	5.7	18.5	2	20	15.6	0.007
6	Fear of getting vaccinated and reason	2	14.8	18	38	24.6	0.023
7	Unable to register	26.3	13	4	20	21.4	0.110
8	Cannot spare a day from work	13	13	6	2	11.5	0.131
9	Vaccination centre is too far	4.7	9.3	6	8	9.5	0.391
10	Timings inappropriate	4.4	9.3	10	6	10.0	0.205
11	No utility of vaccine in containing the pandemic	3.4	7.4	2	22	11.8	0.006
12	Lack of faith in immunization	0	7.4	6	20	11.3	0.000
13	Unable to reach due to physical disability or health condition	10.1	5.6	8	2	8.7	0.011
14	Do not believe in the government system, will get the vaccine once available in the free market/private	0	3.7	0	22	8.7	0.000
15	Lack of supporting documents	14	0	2	2	6.1	0.267
16	Other reasons (fear of overcrowding at the site, fear of symptoms, poor knowledge of process etc.)	0	18.5	0	24	14.4	0.000

AEFI = adverse effects following immunization, COVID-19 = coronavirus disease. ^1^ More than one reason attributed by several responders.

**Table 8:** Description of theme gender-culture with verbatim.

Reasons for NOT Vaccinating	Reasons for Vaccination
Gender-Culture	
*“Often women need to take permissions from their husbands or other family members and are often dependent on their decisions and/opinions to go and get vaccinated.”* *—Key informant, Khadar Delhi* *…distance from the vaccination centre, inappropriate timing at the center and inability to reach the centre due to disability as the reasons for not taking the vaccine yet.* *—A community member, Kolkata*	*“I have shared the information at my home and sisters. I have only accompanied them to get them vaccinated. Even I have shared the information with my neighbours”.* *—A community member, Bangalore*

**Table 9:** Description of theme geographical barrier with verbatim.

Reasons for NOT Vaccinating	Reasons for Vaccination
Geographical Barriers	
*“The centre is too far, regarding the place of access, hospitals are the places to get it, but people are not exactly aware of which hospitals, and there is a very high chance that few clinics might be mistaken for the centre of vaccination.”* *—Govt. Doctor, Key informant, Delhi*	*“My house is adjacent to the government hospital (primary health care centre). When the vaccination drive started, in two or three days even I went and got the vaccine. When I got to know the vaccine was available for people above 45 years of age, I decided to get it.”* *—A Community member, Delhi*

**Table 10:** Description of theme technological barriers with verbatim.

Reasons for NOT Vaccinating	Reasons for Vaccination
Technological Barriers	
*“Registration requires good internet connectivity, digital literacy, both of which are still to penetrate the lower rungs of the society.”* *—Government Doctor, Key informant, Delhi* *“Only those who are well literate, can go to the cyber café or use big mobiles could only avail the facility then. There are people with small mobile, don’t know how to operate them.”* *—Key informant Kolkata*	*“Most of the registrations are done on-site. Very few are online registrations”* *Health worker, Kolkata*,

**Table 11:** Description of theme Socio-economic barrier with verbatim.

Reasons for NOT Vaccinating	Reasons for Vaccination
Socio-Economic Barriers	
*“Yes, people in slum communities, have to go out for work every day. So, at times it’s difficult for them to accompany their aged family members to vaccination centres. Aged family members particularly those above 65–70 need someone to accompany them.”* *—Key informant, Khadar Delhi* *The reasons for vaccination are not religious nor social. They are primarily economic. People are afraid that they will lose their income for a few days at the minimum’* *—A community member, Mumbai*	*“The government needs to push the effectiveness and the need to get vaccinated with the same vigour and strategy as it pushed the polio vaccine campaign.”* *—Community Mobilizer,* *Key informant, Delhi.*

**Table 12:** Description of theme Group influences with verbatim.

Reasons for NOT Vaccinating	Reasons for Vaccination
Group Influences	
*“Even, my daughter cautioned me that nobody would be there to take care of us and I am also weak to get vaccinated. Therefore, I have not undergone vaccination”* —Community member, Bangalore *“Some of the rumours pertained to cases of AEFI. The video of a popular South Indian actor getting a heart attack a day after vaccination has done extensive rounds in Cheeta Camp where several Tamil families live.”* —*A community member, Mumbai*	*All are speaking about this only even my friends told me about it. They talked positively about it and also told us to get (vaccinated) early so that it would be better for us only. Therefore, I went to get the vaccination injection for safety.* —*Community member, Bangalore“I was motivated after watching the news and from hearing about the vaccine since it was important to get rid of this disease. It is important to get vaccinated, no point of being fearful”.* —*A community member, Delhi“My employer convinced me to vaccinate. I decided to get the vaccine to protect myself and my children from COVID-19. My husband also got the vaccine with me.”* —*Community member, Delhi*

**Table 13:** Description of theme individual influences—misinformation/rumor with verbatim.

Reasons for NOT Vaccinating	Reasons for Vaccination
Individual Influences, Mis-Information/Rumours	
*If I can get COVID-19 after the vaccination too, then what’s the point of getting vaccinated. I don’t want to go to risk myself to get complications when there is no certainty.* —*A community member, Madanpur Delhi* *Yes, few people came door to door asking if you are interested. But I don’t want it because I don’t have Corona, only if I get the virus there is some sense in getting the vaccine.* —A community member, Bangalore *‘I was afraid to take the vaccine because I am an asthma patient and I was afraid of how it would impact me. I, therefore, refused to enroll when initially we were told of vaccination for COVID’.* —A community member, Mumbai	*Many people were creating fear in my mind by saying negative side effects which could be possible after getting vaccination injection, they said not to go ahead with it. Even at my home, they were saying no to vaccinations. Therefore, I have been in a panic mood about possible negative side effects. But my manager motivated me to go ahead by consoling me nothing would happen and explained the benefits of safeguarding myself against corona.* —*Community member, Bangalore*

**Table 14:** Description of theme knowledge and awareness with verbatim.

Reasons for NOT Vaccinating	Reasons for Vaccination
AEFI at Individual/Family Level and Trust	
*“I think people still need to know more about the safety and efficacy and more importantly the need of getting vaccinated.”* —*Govt. Doctor, Key informant, Delhi* *A woman in my building died the day after she got vaccinated. This made us really afraid.’* —*A community member, Mumbai* *‘I want to be vaccinated. But my wife is afraid for me. She is stopping me saying what is the hurry? Get vaccinated only after others do’.* —*Key informant, Mumbai*	*“ I have shared the information at my home and sisters. I have only accompanied them to get them vaccinated. Even I have shared the information with my neighbours.”* —*A community member, Bangalore*

**Table 15:** Description of theme experience with past immunization with verbatim.

Reasons for NOT Vaccinating	Reasons for Vaccination
Experience with Past Immunization	
*“The fear among them needs to be addressed by right kind of messaging and that too consistently and continuously. Radio, television, online mediums can be widely used to bust their fears also about aftereffects and the exact need/futility of vaccination. Community volunteers and leaders and NGOs can help in the process, through right channels, without compromising on safety.”* *—Community Mobilizer, Khadar Delhi* *“There were health workers in our group keep informing us about this vaccination, gave us confidence. The people (health workers) had taken vaccination themselves. Plus there was the convenience of getting registered on-site and no queues”* *—A community member, Mumbai*	*“I came to know about it [vaccination drive] from a healthcare worker, then my brother-in-law went and got the vaccine and told me about his experience.”* *—A community member, Delhi*

**Table 16:** Description of theme vaccine supply issues.

Easons for NOT Vaccinating	Reasons for Vaccination
Vaccine Supply Barriers	
*“… How much vaccine will we get? In the last few days, they have given 120, 72 180. Now managing the public is getting difficult. From 3 am, 4 am people are standing in the line.”* *Key informant, Kolkata* *There is no stock of vaccines left. The hospital sent us back saying there is no stock. Once the stock is back we will get vaccinated. Until then if we take care of ourselves there will be nothing to worry about. –* —*A community member, Bangalore* *‘People who even consider getting vaccinated are not sure of waiting time, and availability of vaccines on particular days at particular centres. They, therefore, prefer to wait for a later date.* *—A community member, Mumbai*	*“Despite the budget or issues related to supply, people are willing to come and take vaccine since no other better alternative is available. However, if the supply increases, that will boost vaccine coverage, this will happen soon…”* *—Govt. Doctor, Key informant,* Bangalore.

**Table 17:** Description of theme Access issues with verbatim.

Reasons for NOT Vaccinating	Reasons for Vaccination
Access Barriers	
*I don’t know the schedule, how much time it would take, where to go and what’s the process. I am afraid if I will return vaccinated or not finally* —*A respondent from Madanpur Khadar Delhi* *“We do not know if we go there and will get vaccinated as there are long queues and we are afraid of overcrowding too”.* —*A respondent from Madanpur Khadar, Delhi*	*‘ When people went to Shivaji Nagar centre returned in few hours, it gave me confidence & I went with my son. Seema said ‘I knew in some area people are facing a lot of problem in getting vaccination but for us, we went through a special bus to the centre for vaccination, there was no rush’* *“Easy transportation was available, we did not have to pay any money and everything was convenient, so I went for vaccination’.* —*Community member, Mumbai*

**Table 18:** Description of barriers and enablers to vaccine uptake.

Vaccine Uptake Influences	Barriers and Challenges	Enablers
Contextual influences	Poverty and economic vulnerability resulting in an inability to spare time for vaccination Low digital literacy and poor access to the internet/technology to register for vaccination.	Improved access to vaccination centres through local health facilities, Aam Aadmi Clinics/PHCs, and possibly door to door vaccination drives Facilitating walk-in vaccination and registration
Individual/Group influences	Misinformation on effects of vaccination Poor accessibility for the disabled and elderly population	Increased availability of vaccines at centres Highlighting the importance of vaccination by employers and community leaders
Vaccine specific influences	Uncertainty of vaccine availability Inconvenient timings for vaccination leading to a trade-off between getting vaccinated and a day’s work Lack of proactive communication on the schedule of vaccination drives	Increased knowledge and available information on AEFI Spreading awareness and giving information on vaccine schedule, timing, location

## References

[1] WHO (2020). Vaccines and Immunization. https://www.who.int/health-topics/vaccines-and-immunization#tab=tab_1.

[2] CDC (1999). Ten Great Public Health Achievements—United States, 1900–1999. Morb. Mortal. Wkly. Rep..

[3] GOI (2011). National Vaccine Policy. Ministry of Health & Family Welfare. http://medcontent.metapress.com/index/A65RM03P4874243N.pdf.

[4] Macdonald N. (2015). Vaccine hesitancy: Definition, scope and determinants. Vaccine.

[5] Lin Y., Yen C., Chang Y., Wang P. (2021). Comparisons of Motivation to Receive COVID-19 Vaccination and Related Factors between Frontline Physicians and Nurses and the Public in Taiwan: Applying the Extended Protection Motivation Theory. Vaccines.

[6] WHO (2014). Report of The Sage Working Group on Vaccine Hesitancy.

[7] Eskola J., Duclos P., Schuster M., MacDonald N.E. (2015). How to deal with vaccine hesitancy?. Vaccine.

[8] Revi A., Idicheria C., Jain G., Anand G., Sudhira H.S., Seddon J., Wankhade K., Rashmi M.K., Shetty P., Dhoble R. (2011). Urban India 2011: Evidence.

[9] Roy D., Lees M.H., Palavalli B., Pfeffer K.S. (2014). The emergence of slums: A contemporary view on simulation models. Environ. Model. Softw..

[10] Adiga A., Chu S., Eubank S., Kuhlman C.J., Lewis B., Marathe A., Marathe M., Nordberg E.K., Swarup S., Vullikanti A. (2020). Disparities in spread and control of influenza in slums of Delhi: Findings from an agent-based modelling study. BMJ Open.

[11] Aguilar Ticona J.P., Nery N., Victoriano R., Fofana M.O., Ribeiro G.S., Giorgi E., Reis M.G., Ko A.I., Costa F. (2021). Willingness to get the COVID-19 vaccine among residents of slum settlements. Vaccines.

[12] Ciardi F., Menon V., Jensen J.L., Shariff M.A., Pillai A., Venugopal U., Kasubhai M., Dimitrov V., Kanna B., Poole B.D. (2021). Knowledge, Attitudes and Perceptions of COVID-19 Vaccination among Healthcare Workers of an Inner-City Hospital in New York. Vaccines.

[13] Nohl A., Afflerbach C., Lurz C., Brune B., Ohmann T., Weichert V., Zeiger S., Dudda M. (2021). Acceptance of COVID-19 Vaccination among Front-Line Health Care Workers: A Nationwide Survey of Emergency Medical Services Personnel from Germany. Vaccines.

[14] WHO (2019). Ten Threats to Global Health in 2019.

[15] Schuster M., Eskola J., Duclos P., Group W. (2015). Review of vaccine hesitancy: Rationale, remit and methods. Vaccine.

[16] Viswanath K., Bekalu M., Dhawan D., Pinnamaneni R., Lang J., McLoud R. (2021). Individual and social determinants of COVID-19 vaccine uptake. BMC Public Health.

[17] Luo H., Qu H., Basu R., Rafferty A.P., Patil S.P., Cummings D.M. (2022). Willingness to Get a COVID-19 Vaccine and Reasons for Hesitancy Among Medicare Beneficiaries: Results from a National Survey. J. Public Health Manag. Pract..

[18] Khaled S.M., Petcu C., Bader L., Amro I., Al-Hamadi A.M.H., Al Assi M., Ali A.A.M., Le Trung K., Diop A., Bellaj T. (2021). Prevalence and Potential Determinants of COVID-19 Vaccine Hesitancy and Resistance in Qatar: Results from a Nationally Representative Survey of Qatari Nationals and Migrants between December 2020 and January 2021. Vaccines.

[19] Echoru I., Ajambo P.D., Keirania E., Bukenya E.E.M. (2021). Sociodemographic factors associated with acceptance of COVID-19 vaccine and clinical trials in Uganda: A cross-sectional study in western Uganda. BMC Public Health.

[20] Akwataghibe N.N., Ogunsola E.A., Broerse J.E., Popoola O.A., Agbo A.I., Dieleman M.A. (2019). Exploring Factors Influencing Immunization Utilization in Nigeria—A Mixed Methods Study. Front. Public Health.

[21] Bogart L.M., Dong L., Gandhi P., Klein D.J., Smith T.L., Ryan S., Ojikutu B.O. (2021). COVID-19 Vaccine Intentions and Mistrust in a National Sample of Black Americans. J. Natl. Med. Assoc..

[22] Bono S.A., Faria de Moura Villela E., Siau C.S., Chen W.S., Pengpid S., Hasan M.T., Sessou P., Ditekemena J.D., Amodan B.O., Hosseinipour M.C. (2021). Factors Affecting COVID-19 Vaccine Acceptance: An International Survey among Low- and Middle-Income Countries. Vaccines.

[23] Han K., Francis M.R., Zhang R., Wang Q., Xia A., Lu L., Yang B., Hou Z. (2021). Confidence, Acceptance and Willingness to Pay for the COVID-19 Vaccine among Migrants in Shanghai, China: A Cross-Sectional Study. Vaccines.

[24] Trabucco Aurilio M., Mennini F.S., Gazzillo S., Massini L., Bolcato M., Feola A., Ferrari C., Coppeta L. (2021). Intention to Be Vaccinated for COVID-19 among Italian Nurses during the Pandemic. Vaccines.

[25] Babicki M., Mastalerz-Migas A. (2021). Attitudes toward Vaccination against COVID-19 in Poland. A Longitudinal Study Performed before and Two Months after the Commencement of the Population Vaccination Programme in Poland. Vaccines.

[26] To Q.G., Stanton R., Khalesi S., Williams S.L., Alley S.J., Thwaite T.L., Fenning A.S., Vandelanotte C. (2021). Willingness to Vaccinate against COVID-19 Declines in Australia, Except in Lockdown Areas. Vaccines.

[27] Wawrzuta D., Jaworski M., Gotlib J. (2021). What Arguments against COVID-19 Vaccines Run on Facebook in Poland: Content Analysis of Comments. Vaccines.

[28] Butler R., Macdonald N.E., Group W. (2015). Diagnosing the determinants of vaccine hesitancy in specific subgroups: The Guide to Tailoring Immunization Programmes (TIP). Vaccine.

[29] Alabdulla M., Reagu S.M., Al-Khal A., Elzain M., Jones R.M. (2021). COVID-19 vaccine hesitancy and attitudes in Qatar: A national cross-sectional survey of a migrant-majority population. Influenza Other Respir. Viruses.

[30] Doherty I., Pilkington W., Brown L., Billings V., Hoffler U., Paulin L., Kimbro S.K., Baker B., Zhang T., Locklear T. (2021). COVID-19 Vaccine Hesitancy in Underserved Communities of North Carolina. medRxiv.

[31] Abedin M., Islam M.A., Rahman F.N., Reza H.M., Hossain M.Z., Hossain M.A., Arefin A., Hossain A. (2021). Willingness to vaccinate against COVID-19 among Bangladeshi adults: Understanding the strategies to optimize vaccination coverage. PLoS ONE.

[32] Loomba S., de Figueiredo A., Piatek S.J., de Graaf K., Larson H.J. (2021). Measuring the impact of COVID-19 vaccine misinformation on vaccination intent in the UK and USA. Nat. Hum. Behav..

[33] Arce J.S.S., Warren S.S., Meriggi N.F., Scacco A., McMurry N., Voors M., Syunyaev G., Malik A.A., Aboutajdine S., Armand A. (2021). COVID-19 vaccine acceptance and hesitancy in low- and middle-income countries. Nat. Med..

[34] Alizadeh M., Abbasi M., Bashirivand N., Mojtahed A., Karimi S.E. (2020). Nongovernmental organizations and social aspects of COVID-19 pandemic: A successful experience in health policy. Med. J. Islamic Repub. Iran.

[35] Malik M.N., Awan M.S., Saleem T. (2020). Social Mobilization Campaign to Tackle Immunization Hesitancy in Sargodha and Khushab Districts of Pakistan. J. Glob. Health.

[36] Puri N., Coomes E.A., Haghbayan H., Gunaratne K. (2020). Social media and vaccine hesitancy: New updates for the era of COVID-19 and globalized infectious diseases. Hum. Vaccines Immunother..

[37] Naniche D., Hotez P., Bottazzi M.E., Ergonul O., Figueroa J.P., Gilbert S., Gursel M., Hassanain M., Kang G., Kaslow D. (2021). Beyond the jab: A need for global coordination of pharmacovigilance for COVID-19 vaccine deployment. EClinicalMedicine.

[38] Christopher S., Watts V., McCormick A.K.H.G., Young S. (2008). Building and maintaining trust in a community-based participatory research partnership. Am. J. Public Health.

[39] WHO (2020). Managing the COVID-19 Infodemic: Promoting Healthy Behaviours and Mitigating the Harm from Misinformation and Disinformation.

[40] Burgos R.M., Badowski M.E., Drwiega E., Ghassemi S., Griffith N., Herald F., Johnson M., Smith R.O., Michienzi S.M. (2021). The race to a COVID-19 vaccine: Opportunities and challenges in development and distribution. Drugs Context.

[41] Press V.G., Huisingh-Scheetz M., Arora V.M. (2021). Inequities in Technology Contribute to Disparities in COVID-19 Vaccine Distribution. JAMA Health Forum.

[42] Mohamoud S.A., Ali M.A., Muse A.M., Bile A.S., Mohmud A.J. (2021). COVID-19 Vaccine Rollout in Somalia: Experiences and Challenges in Fragile Context. https://www.africaportal.org/publications/covid-19-vaccine-rollout-somalia-experiences-and-challenges-fragile-context/.

[43] Walker D.M., Yeager V.A., Lawrence J., Mcalearney A.S. (2021). Identifying Opportunities to Strengthen the Public Health Informatics Infrastructure: Exploring Hospitals’ Challenges with Data Exchange. Milbank Q..

[44] Nidirect (2021). ‘Big Jab Weekend’ will Boost Vaccination Drive.

[45] World Health Organization, UNICEF (2021). Disability Considerations for COVID-19 Vaccination.

